# Identifying Barriers to Implementing Pain Management for Piglet Castration: A Focus Group of Swine Veterinarians

**DOI:** 10.3390/ani10071202

**Published:** 2020-07-15

**Authors:** Brooklyn Wagner, Kenneth Royal, Rachel Park, Monique Pairis-Garcia

**Affiliations:** 1 Department of Population Health and Pathobiology, College of Veterinary Medicine, North Carolina State University, Raleigh, NC 27606, USA; rmpark@ncsu.edu (R.P.); pairis-garcia@ncsu.edu (M.P.-G.); 2 Department of Clinical Sciences, College of Veterinary Medicine, North Carolina State University, Raleigh, NC 27606, USA; kdroyal2@ncsu.edu

**Keywords:** pig, piglet, drug, pain mitigation, NSAID

## Abstract

**Simple Summary:**

Surgical castration is a painful husbandry procedure typically performed on piglets in the United States (US) within the first week of life. Castration is used to improve meat quality, and as a result, nearly all male pigs destined for slaughter in the US will be castrated. In recent years, consumers and retailers have questioned the ethicality of castration as a production practice, given that it results in pain experienced by the piglet. However, eliminating castration is not practical at this time in the US, and the adoption of pain management protocols remains the most viable solution to managing pain associated with castration. Given that veterinarians often have direct oversight regarding the development of animal care protocols, the objective of the present study is to identify factors influencing swine veterinarian decision-making in regard to pain management for piglet castration using focus group methodologies. Three main areas of focus were identified and included (1) the lack of approved products validated for efficacy, (2) economic limitations and challenges, and (3) deficient guidelines and training for veterinarians to develop protocols. These barriers must be addressed, moving forward, to support the use of pain management protocols for castrated piglets throughout the US swine industry.

**Abstract:**

Surgical castration is a painful husbandry procedure performed on piglets in the United States (US) to improve meat quality. Veterinarians play a crucial role in developing pain management protocols. However, providing pain management for castration is not common practice in US swine production systems. Therefore, the objective of the present study is to identify factors influencing swine veterinarian decision-making in regard to pain management protocols for piglet castration using focus group methodologies. Swine veterinarians (n = 21) were recruited to participate in one of three focus groups. Audio recordings were transcribed verbatim and analyzed by two independent coders who identified three areas of focus, including (1) the lack of approved products validated for efficacy, (2) economic limitations and challenges, and (3) deficient guidelines and training for veterinarians to develop protocols. Although participating veterinarians acknowledged the importance of pain management from an animal welfare standpoint, these barriers must be addressed to ensure that castration pain can be successfully mitigated on-farm.

## 1. Introduction

Surgical castration is a painful husbandry procedure typically performed on piglets in the United States (US) within the first week of life [1,2,3]. Castration is performed to improve meat quality through the elimination of boar taint, an undesirable odor present in the meat of intact males [4]. As a result, nearly all male pigs destined for slaughter in the US will be castrated [5].

In recent years, consumers and retailers have questioned piglet castration as a production practice, given that it results in pain experienced by the pig [6]. These concerns have resulted in the development and implementation of strategies that aim to mitigate or eliminate castration pain entirely [7,8,9,10,11]. However, even when policies and legislation are enacted to support the universal adoption of on-farm pain management protocols for piglets at castration, enforcement is challenging. For example, in 2010, the “European Declaration on alternatives to surgical castration of pigs” was agreed upon, stipulating that by 2012, piglet castration can only be performed with prolonged analgesia and/or anesthesia, and by 2018, surgical castration should be phased out altogether. However, in a survey conducted in 2016 by De Briyne and colleagues, more than 80% of male pigs were still castrated, and only 5% of this castrated population received anesthesia and analgesia [11]. This unresolved discrepancy continues to represent an animal welfare challenge for swine production on a global level. 

Data prevalence for US swine farms is lacking but is likely to have a similar prevalence of castrated pigs in the EU, with a lower prevalence of analgesia/anesthetic use [5]. Such discrepancies may be even more extensive in the US than initially explicated, impacting an estimated 94 million piglets annually. To date, the US maintains no state or federal legislation requiring that pain relief be provided to piglets at castration. In addition, approval from the Food and Drug Administration (FDA) is required in order for producers or caretakers to administer such pain relief drugs without oversight by a veterinarian. Given these challenges, pain management protocols vary across the US swine industry, and remain largely underexamined, when compared with fellow pork-producing countries. 

Within the swine industry, veterinarians play a crucial role in implementing pain management protocols on-farm. Veterinarians are often responsible for direct oversight regarding the development and execution of animal care protocols. In addition, veterinarians are viewed by the public as animal welfare stewards [12,13], particularly in regard to alleviating pain and suffering. However, in the US, swine veterinarians do not have access to an FDA-approved drug for pain relief in swine [14]. In fact, swine veterinarians can only prescribe pharmaceuticals regulated under the Animal Medicinal Drug Use Clarification Act of 1994 to mitigate pain in an extralabel manner [15]. Although this option is available, implementing pain management protocols for castration is not common practice in US swine production systems [16]. Therefore, additional barriers are likely impacting, and ultimately impeding, the use of pain management for piglets at castration. Therefore, the objective of the present study is to identify factors influencing swine veterinarian decision-making in regard to pain management protocols for piglet castration using focus group methodologies.

## 2. Materials and Methods 

All research was reviewed and approved by the North Carolina State University Institutional Review Board Committee for Human Subjects Research (Protocol #20299).

### 2.1. Participant Recruitment and Focus Group Format

Swine veterinarians attending the March 2020 American Association of Swine Veterinarians (AASV) Annual Meeting were targeted for this study. Focus groups were held at this conference, given the large number of swine veterinarians that attend the meeting on an annual basis. The AASV was charted in 1969 and is based out of Perry IA, with approximately 1548 members, 309 of which are students. These members are involved in practice, industry, and academia specific to the swine industry and are from more than 40 countries; thus, veterinarians from outside of the US were given the opportunity to participate. With support from the conference organizers, a recruitment advertisement was sent to AASV members via the AASV weekly e-letter to recruit participation in one of three focus groups. All members of the AASV have access to the e-letter; therefore, the opportunity to participate was provided to all AASV members who read the e-letter. Information regarding the focus groups was included in 10 e-letters from 15 November 2019 to 5 February 2020. To encourage participation, a $100 VISA gift card was provided to any individual who participated in the focus groups. Interested individuals were required to sign a consent form prior to participation. To promote confidentiality, participants were encouraged to maintain the privacy of other focus group participants and related discussions. The sample size was justified by conducting focus groups until data saturation was reached (i.e., no new information could be obtained) [17,18].

Demographic information was collected prior to the start of focus group discussion and included gender, age, race, current role, and the number of years engaged in the swine industry and total sow inventory under direct oversight. Discussions were prompted using six predetermined base questions (Table 1) asked by one trained moderator to promote consistency and eliminate any effects of interindividual variability. In addition, eight predetermined follow-up questions could also be used to expand discussion points at the discretion of the moderator. Moderator training was provided through the Collaborative Institution Training Initiative (CITI). This program is “dedicated to promoting the public’s trust in the research enterprise by providing high quality, peer-reviewed, web-based education courses in research, ethics, regulatory oversight, responsible conduct of research…and other topics” [19]. The moderator was required to complete training programs and receive a passing score of 80% before being eligible to moderate the focus groups. 

### 2.2. Discussion Analysis

Audio recordings of focus group discussions were transcribed verbatim. Two researchers used a grounded theory [20], qualitative data approach for analysis. Coder 1 is an associate professor with a PhD in measurement and evaluation. Coder 2 is a postdoctoral research scholar in animal welfare with a PhD and expertise in animal production and physiology. 

An emergent–systematic focus group design was employed, in which the first group served as the exploratory group, and the other two groups served a combination purpose of exploration and verification [21,22]. Constant comparison analysis techniques were used to assess saturation across the three groups. Open coding, a process in which each transcript was read by each researcher independently, and key words and phrases were identified and noted, was utilized on coded data. For example, the statement “…if it’s a requirement for the farm to do it, they will provide the labor. And it would increase cost…at some point, you have to get paid back for your investment,” generated the following codes: “Protocol/Procedure”, “Time/Labor”, and “Economics”. From open coding, selective coding was employed to provide richer dimensions to the research problem by grouping codes together into categories. For example, “Economics” and several other codes formed an area of focus categorized as “Logistical Factors”. Researchers then discussed and compared results to ensure that common conceptualizations of the data occurred. Next, selective coding, in which the researchers developed areas of focus that emerged based on the synthesized data, was conducted. 

## 3. Results

### 3.1. Demographics

Twenty-one swine veterinarians participated in one of three focus groups (7, 8, and 6 participants/group, respectively; n = 21). A majority (66.7%) of focus group participants were male and indicated their race as “White” (95.2%). Participant age, years of experience, and primary role within the swine industry are presented in Table 2. The median number of sows in which the participants had direct oversight was 2000 (ranging between 80 to 1,000,000 sows). 

### 3.2. Focus Group Discussion

When asked “*What comes to mind when you think about castrating piglets on a farm?*”, the majority of participants noted that, although controversial, it is an important and necessary practice. Others noted that piglet castration as an economically driven process is time-consuming and labor-intensive. Several participants also noted that the procedure is both risky and stressful for the animal. 

When asked “*What are the main reasons to provide pain relief to piglets for castration?*”, the participants primarily stated that it improves animal welfare and wellbeing. Numerous participants noted the provision of pain relief to piglets at castration is the “right” thing to do from an ethical perspective and indicated that it has become a consumer expectation. In addition, the participants even noted that providing pain relief may send a positive message to caretakers and emphasize that animal welfare is taken seriously. Others highlighted the potential benefits to pig health and productivity. 

When asked “*What are the main reasons to NOT provide pain relief to piglets for castration?*”, most participants stated the primary reasons as the lack of proven benefit and the fact that there are currently no pain relief products approved for use in piglets in the US. Furthermore, proving drug efficacy, and subsequently obtaining approval, remains difficult because measurements of pain have not been validated. The participants also noted that there are no specific guidelines that include size or age considerations. Other participants stated that providing pain relief is a labor-intensive practice without a worthwhile return on investment, with one participant specifically noting that there are currently no financial benefits from packers or retailers to justify the additional operating cost. Several participants noted the importance of minimizing the number of handling events and mentioned concerns regarding the potential for unintended consequences (e.g., side effects of the drug, greater injury risk to the piglets while medicated). 

When asked “*What other factors might you consider when making the decision to implement a protocol that involves providing pain relief for castration on-farm?*”, the participants expressed a wide range of responses. Several participants noted being uncertain if veterinarians or farm staff would be tasked with providing oversight should pain relief protocols be implemented for castration on-farm. A need for training (e.g., drug administration) and the associated costs were also noted as challenges within focus group discussions. The participants particularly noted the challenges associated with demonstrating productivity and economic benefits, as well as ensuring caretaker safety. In addition, withdrawal times and food safety considerations were also discussed. Several participants noted potential issues with cross-compatibility with other products administered concurrently and the need to base decisions on outcome-based parameters. Several participants again noted the paramount need for drug efficacy to be established and the duration of pain relief effects to be defined. Lastly, several participants also stated a need for piglet-specific dosage guidelines.

## 4. Discussion

Animal pain is an aversive sensory experience that results in both physiological and behavioral changes to the animal [23]. Prevention and mitigation of pain and suffering in animals is an ethical obligation for veterinarians, as demonstrated by the Veterinarians’ Oath taken upon entering the profession (“*Being admitted to the profession of veterinary medicine, I solemnly swear to use my scientific knowledge and skills for the benefit of society through the protection of animal health and welfare, the prevention and relief of animal suffering, the conservation of animal resources, the promotion of public health, and the advancement of medical knowledge...”* [24]). Castration is a common production practice implemented on-farm across multiple species (e.g., pigs, cattle, small ruminants) and is considered painful based on physiological and behavioral responses indicative of pain [5]. However, pain mitigation is not routinely implemented for piglets undergoing castration in commercial operations. Given that veterinarians play a key role in the development of on-farm protocols to manage pain, understanding the barriers to implementing pain management is a key step to ensuring pain control for castration in the future. Therefore, the objective of the present study is to identify factors influencing swine veterinarian decision-making in regard to pain management protocols for piglet castration using focus group methodologies.

Castration-associated pain adversely affects the individual pig’s affective state and can negatively impact behavior and productivity [25]. Veterinarians serve as the primary resource in educating, training, and supporting producers with implementing pain management on-farm. For swine veterinarians, opportunities to mitigate pain include developing pharmaceutical protocols to manage pain associated with common production practices such as castration. However, observational experiences in the industry suggest it is uncommon for pain management to be provided before or during the castration procedure in the US, and there are no studies to date that provide prevalence data on pain management practices for castration in the US [16,26]. Therefore, the objective of the present study is to identify factors influencing swine veterinarian decision-making in regard to pain management protocols for piglet castration using focus group methodologies.

In contrast to the legislative approach used in Canada and the European Union [10,27], the US has addressed the need for pain management as a result of policy development by retailers in response to concerned citizens and consumers [28,29]. Veterinarians do and will continue to play a key role in highlighting the importance of using appropriate production practices that optimize animal welfare in livestock species [30]. In the present study, participating veterinarians considered pain management to be a critical tool for ensuring good welfare for pigs on-farm. Numerous participants voiced their belief that managing castration pain was the “right thing to do” and postulated that implementing pain management demonstrates the importance of animal welfare, to the veterinarian and/or the company, from a caretaker’s perspective. One participating veterinarian stated that “…providing pain relief to the animals sends the right message…we spend a lot of time talking about them treating the animals right and we ask them to do this painful procedure that they know has to hurt…If we really want them to embrace the ideals we think are appropriate, we should be modeling that if possible, if the options are out there.” 

In addition, several participants highlighted the potential benefits to pig health and productivity. For example, one participant stated that an effective product would involve physiological improvements such as “…quicker nursing, better weight gains post-castration…”, while another highlighted the action behind pain relief drugs, stating that “…a lot of NSAIDs [nonsteroidal anti-inflammatory drugs] could potentially help in the different pathways of inflammation… [leading] to the piglet coming back to normal behavior and potential performance”. Results from this study are in agreement with previous work addressing veterinarian attitudes towards pain management. A study surveying veterinarians in the UK reported that veterinarians believe that providing pain relief allows animals to recover more quickly and view the addition of pain relief as a positive outcome for both animals and farmers [31]. 

Although participating veterinarians acknowledged the importance of pain management from an animal welfare standpoint, the participants also voiced significant concerns over implementing pain management for castration. Three main areas of focus that were consistent across all focus groups and specific to barriers to pain management implementation were identified and included (1) the lack of approved products validated for efficacy, (2) economic limitations and challenges, and (3) deficient guidelines and training for veterinarians to develop protocols. 

Objectively evaluating pain in livestock species remains a challenge due to variations in individual animal pain perception [32], as well as procedure-dependent differences in pain sensitivity [33]. Quantifying pain associated with castration has been primarily conducted by evaluating behavioral and physiological responses to the procedure, including changes in cortisol, β-endorphins, vocalizations, and pain-related behavioral endpoints [1,25]. Although there are well over fifty publications that evaluate castration pain in pigs, no studies to date have validated these endpoint measurements used to quantify castration pain [1,2,34]. When recommending pharmaceutical intervention for pain, the lack of validated measures, combined with poor-quality evidence demonstrating drug efficacy, has prevented the development of nationally accepted industry guidelines for pain management [2]. 

Veterinarians participating in this focus group were hesitant to provide pain relief during castration without strong scientific evidence of drug efficacy, as indicated by an FDA-approved label. One veterinarian stated, “Product approval has been a huge detriment to our industry in getting something done about pain mitigation”. Another veterinarian added, “One of the challenges is finding pain medication that’s truly effective…and today, we’re lacking data to make those decisions…or we don’t have an approved product”. To date, the US has no FDA-approved drugs labeled for pain control in pigs. In order to achieve FDA-label approval, methods used to assess pain and drug efficacy must be well-defined and validated [35]. Future work is needed to develop a research protocol utilizing well-defined, reliable endpoints that measure castration pain in piglets. Preliminary work conducted by the Pain Mitigation Assessment Protocol Consortium, funded through the National Pork Board and Iowa Pork Producers Association, has begun the first steps in this process. This consortium, composed of individuals with expertise in assessing pain in swine, was established, and objectives are underway to validate behavioral, physiologic, and biomarker endpoints that reliably measure pain associated with surgical castration in neonatal piglets [35].

In addition to the absence of an FDA-approved product for managing castration pain in piglets, economic constraints were also identified as a major barrier to implementing pain management. For example, one veterinarian stated, “Cost. I mean, this is important. I want to do it, but I’m not gonna go bankrupt trying so...”, and another specified, “You’d have to offset that cost…when a new process comes up, you have to evaluate it…”. 

**Figure 1:**
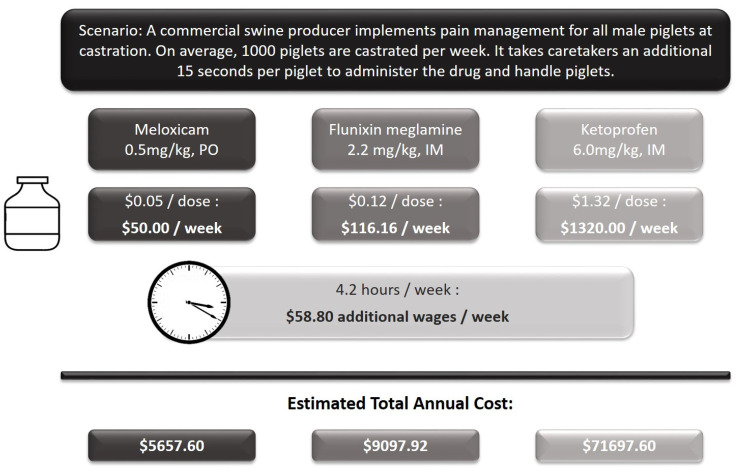
An example scenario of pain management implementation costs (presented as US dollars).

Flunixin meglumine, meloxicam, and ketoprofen are three NSAIDs that have been studied most frequently in regard to castration pain mitigation [16,26,36,37]. On a per-piglet basis, each of these drugs yields a treatment cost of approximately $0.05 to $1.32 per piglet. In addition to the drug cost, labor and equipment costs needed to administer the drug would need to be taken into account as well. A detailed example and breakdown of the additional costs can be found in Figure 1.

These additional costs, coupled with the lack of proven health and performance benefits that may help offset such costs, make it difficult for veterinarians to make a strong, economically grounded argument for producers to implement pain management protocols [38]. These results are in agreement with research conducted by Hewson and colleagues [39] who surveyed Canadian swine veterinarians’ attitudes towards pain management. In this Canadian study, veterinarians noted a lack of cost-effective drugs available for livestock and voiced concerns regarding additional costs associated with professional time spent implementing pain management on-farm [39]. It is therefore unlikely that in the US, pain management will be driven by economic benefits for the producer, and ethical considerations and consumer demand will likely be the driving force [40]. 

Producers rely heavily on their veterinarians to develop farm-specific protocols for pain management [40]. This relationship can be beneficial as the producer can implement effective pain management with a protocol that clearly outlines the process for caretakers responsible for administering the drug. However, pain management protocols must be scientifically-validated and include information specific to dosage, administration route, and frequency. To date, veterinarians have limited access to science-based resources to help guide protocol development and select the appropriate pharmaceutical intervention to manage pain specific to piglet age and/or size. For example, one veterinarian stated, “I haven’t seen any data that has demonstrated to me, with any degree of comfort, about what I am supposed to be using for a baby pig. The products that are licensed are for mature, large animals, and the metabolism in a piglet is different.” Another expressed frustration regarding the limitations of available pharmaceuticals, specifically stating, “…is it at a concentration that’s formulated to give to such a small bodyweight…injectable, oral, topical applications…can you get that into a volume that would work for the size of animal that we actually wanna give it to”. Irrespective of drug approval status, veterinarians consistently discussed deficiencies in drug-use guidelines currently available to direct protocol development.

In addition to protocols, interactive training tools may be beneficial to the industry to help swine caretakers understand the importance of pain management. Interactive training tools are often more effective for caretakers [41,42] as they do not rely heavily on text to deliver complex information or use high-level technical jargon that is confusing to caretakers [43,44]. Therefore, future work is needed to provide science-based information to support veterinarians in developing effective pain management protocols that are specific to the procedure and the piglet.

This study effectively highlights some of the major challenges perceived by veterinarians in regard to implementing pain management for piglets undergoing castration. However, results from this study may be biased, given that veterinarians who participated in the focus groups are likely to be educated on this topic given their interest in participating and may represent a more proactive subpopulation of swine veterinarians. Future work is needed to recruit a larger population of swine veterinarians to encompass all perceived barriers to pain management.

## 5. Conclusions

In conclusion, managing pain specific to castration is viewed importantly by veterinarians in regard to safeguarding animal welfare. However, the lack of an approved product for controlling piglet pain, coupled with economic challenges and insufficient science-based guidelines, makes it challenging for swine veterinarians to advocate for and develop pain management protocols for castration on US swine farms. Validation of physiological and behavioral endpoints to quantify pain is the first step needed to obtain FDA approval for a drug specific for pain relief in piglets. In addition, development and access to science-based guidelines in which veterinarians can use to develop pain management protocols is needed to ensure pain associated with castration can be successfully managed and mitigated. 

## Figures and Tables

**Table 1:** Primary (Q) and follow-up (F) questions utilized in focus group discussions on piglet castration and pain relief on swine farms in the United States.

Question Type #	Question
Q1	What comes to mind when you think about castrating piglets on-farm?
Q2	What are the main reasons to provide pain relief to piglets for castration?
Q3	What, if any, are the additional benefits of providing pain relief to piglets for castration?
Q4	What are the main reasons to NOT provide pain relief to piglets for castration?
Q5	What, if any, are the additional drawbacks to providing pain relief to piglets for castration?
Q6	What other factors might you consider when making the decision to implement a protocol that involves providing pain relief for castration on-farm?
F1	Can you say more about what you have just told me?
F2	Can you elaborate on that statement?
F3	You mentioned [*brief summary of participant statement*]. Can you discuss this point further?
F4	Can you give me an example of this issue?
F5	What do you think consumers would say if they found out piglets were/were not provided pain relief for castration? *(for questions 3 and 5)*
F6	Are there benefits/drawbacks for caretakers that provide pain relief to piglets for castration? *(for questions 3 and 5)*
F7	Are there economic benefits/drawbacks? *(for questions 3 and 5)*
F8	What do you think the legal considerations are for providing pain relief to piglets for castration? *(for questions 3 and 5)*

**Table 2:** Focus group participant demographic information.

Question	Response Options	Participant #
What is your age?	29 years or younger	2
	30–39 years	7
	40–49 years	4
	50–59 years	2
	60 years or older	6
What is your current primary role in the swine industry?	(Practitioner) Veterinarian working in private practice with or without ownership of pigs	5
	(Practitioner) Veterinarian working within production systems	5
	(Public/Corporate) Veterinarian working in academia	5
	(Public/Corporate) Veterinarian working within the allied pork industry	3
	Other, please specify *(text line provided)*	3
How many years have you been involved with the swine industry?	1 year	0
	2–5 years	2
	6–10 years	6
	11–15 years	3
	16–20 years	2
	21–25 years	0
	Over 25 years	8
